# The Phantom Urine: An Unexpected Finding during a Routine Cesarean Section

**DOI:** 10.1155/2014/575032

**Published:** 2014-02-11

**Authors:** Maria Pagnozza, Chahin Achtari, Jean-Yves Meuwly, David Baud

**Affiliations:** ^1^Department of Obstetrics and Gynecology, University Hospital, 1011 Lausanne, Switzerland; ^2^Department of Radiology, University Hospital, 1011 Lausanne, Switzerland; ^3^Materno-Fetal and Obstetrics Research Unit, University Hospital, 1011 Lausanne, Switzerland

## Abstract

We present here an atypical finding during an elective repeat cesarean section. Despite urine flow through an indwelling bladder catheter, bladder remains distended during the whole procedure. Unexpected anatomical variations and malformations can make routine surgery challenging. Urinary tract anomalies should be suspected in cases of unexpected difficult bladder catheterization.

## 1. Introduction

Maternal malformation can be diagnosed during pregnancy with ultrasound imaging. However, most of them remain undiagnosed or diagnosed unexpectedly during routine surgery. We present here a challenging repeat cesarean section with an unusual urinary finding.

## 2. Case Report

A 36-year-old G3P2 patient with a history of two previous caesarean sections was admitted for an elective repeat cesarean section at 39 weeks of gestation. The routine preprocedure preparation was uneventful and an indwelling bladder catheter was placed without difficulty. Upon peritoneal entry, the bladder was noted to be significantly distended despite the fact that urine was confirmed in the urinary drainage bag. In an attempt to empty the bladder, a new 12 G Foley catheter was placed, followed by a rigid silicone catheter, but the bladder remained distended. In order to have a better view of the pelvic organs, fetal extraction and uterotomy closure were decided.

Persistence of bladder distension might have resulted from a urinary tract injury at initial catheterization. To investigate this hypothesis, 400 mL of methylene blue was injected through the urinary catheter. The bladder volume did not change and no methylene blue was noted intraabdominally. After this negative test, most of the 400 mL of methylene blue flowed back into the urinary bag and the remaining blue-colored urine came in intermittent streams. A rectal digital examination did not reveal any injuries.

**Figure 1:**
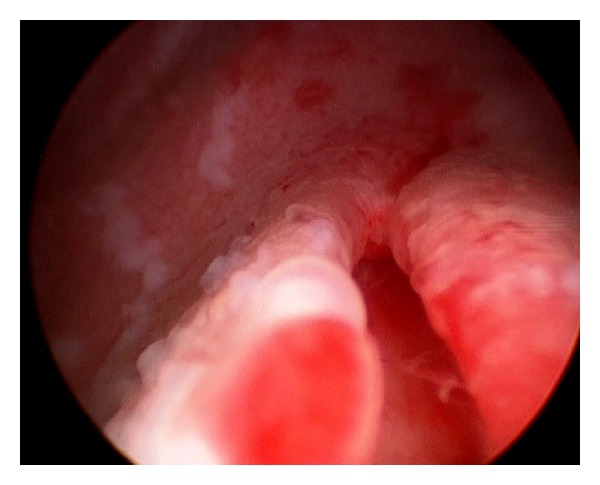
Intraoperative cystoscopy picture showing a fold on the posterior wall of the bladder neck.

Cystoscopy confirmed a distended bladder and two patent ureteral meatus. No trace of methylene blue was identified within the bladder. At the end of the cystoscopy procedure, a fold was noted on the posterior wall of the bladder neck (see Figure 1). This fold was initially thought to be iatrogenic. Insertion of the cystoscope inside this fold revealed a dilated peristaltic tubular structure containing methylene blue. This finding confirmed that the urinary catheter entered this ectopic ureter, and not the bladder. In order to avoid this fold and empty the bladder, a urinary catheter was placed under direct visualization. The postoperative course was uneventful and antibiotic prophylaxis was administered for 48 hours.

**Figure 2:**
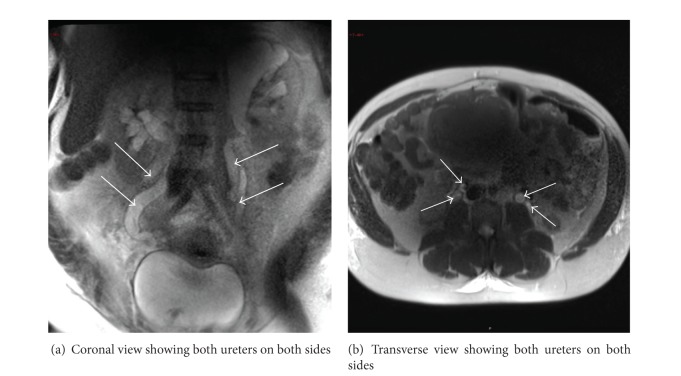
Abdominal Magnetic resonance imaging picture of the patient at day 2 post cesarean section.

Magnetic resonance imaging showed a left ureteral duplication with chronic renal pelvic dilatation of the superior calyceal system and associated cortical atrophy. The right kidney had a proximal bifid ureter (see Figure 2).

## 3. Discussion

Ureteral duplication, whether partial or complete, is the most common congenital anomaly of the urinary tract with an incidence of 0.8–5% [1, 2]. However, this might be underestimated, since affected individuals are usually asymptomatic. The diagnosis is made either during autopsy [1] or incidentally during radiological examinations [3]. Although renal and urinary tract anomalies represent 20–30% of the anomalies identified prenatally, there is an improvement in detection with the widespread availability of antenatal ultrasound screening.

Ureteral duplication is suspected when the ureter is located caudal to its normal insertion site on the bladder's trigone. This malformation is usually associated with a duplex collecting system, resulting from the duplication of the ureteric bud that arises from the mesonephric duct [1]. In our case, one of the ureters joined the bladder at its normal expected location, whereas the ectopic ureter was inserted into the bladder neck.

Ectopic ureters were commonly inserted in the bladder neck or upper urethra (33%), vaginal vestibule (33%), vagina (25%), or the cervix or uterus (<5%) [1]. In 80% of cases, these defects are associated with a double collecting system and a duplex kidney [4]. Embryologically, cranial and caudal ureters are associated with lower and upper poles of the kidney, respectively. The upper part of the kidney is often hypoplastic or dysplastic.

Only patients with the ureteric orifice located on the bladder neck or urethra are continent. In the other cases, incontinence might be difficult to recognize, since persistent leaking or moisture might be considered normal by the patient. These patients are at an increased risk of urinary tract infections due to ureteral dilatation and urinary stasis.

Urinary tract anomalies should be suspected in cases of unexpected difficult bladder catheterization. Since congenital anomalies of the kidney and urinary tract play a role in 30–50% of cases of end stage renal disease, it is essential to identify these anomalies in order to prevent progressive kidney damage. Although urinary catheterization is controversial for some investigators [5], its absence can make a cesarean section challenging.
